# New Lobane and Cembrane Diterpenes from Two Comorian Soft Corals

**DOI:** 10.3390/md8020359

**Published:** 2010-02-23

**Authors:** Isabelle Bonnard, Sabina B. Jhaumeer-Laulloo, Nataly Bontemps, Bernard Banaigs, Maurice Aknin

**Affiliations:** 1 Laboratoire de Chimie des Biomolécules et de l’Environnement, Centre de Phytopharmacie, Université de Perpignan, 52 avenue Paul Alduy, 66860 Perpignan Cedex, France; 2 Department of Chemistry, University of Mauritius, Réduit, Mauritius; E-Mail: sabina@uom.ac.mu; 3 Laboratoire de Chimie des Substances Naturelles et des Sciences des Aliments, 15, Faculté des Sciences et Technologies, Université de La Réunion, avenue René Cassin-BP 7151, 97715 Saint Denis, Messag., Cedex 9, France; E-Mail: maurice.aknin@reunion.iufm.fr

**Keywords:** lobane, cembrane, acetylcholinesterase inhibitors, Sarcophyton, Lobophytum

## Abstract

Preliminary biological investigation of a collection of Comorian soft corals resulted in the selection of two specimens, one of *Sarcophyton* and the other of *Lobophytum,* on the basis of their toxicity on larvae of the brine shrimp *(Artemia salina)* and inhibition of acetylcholinesterase, respectively. Bioassay-guided fractionations provided a known antitumor promoter cembrane diterpenoid, (+)-sarcophytol-A (**1**), along with a new lobane diterpenoid, carbomethoxyfuscol (**2**), from *Sarcophyton* sp., and a new cembranoid, crassumolide E (**3**), from *Lobophytum* sp. The structures of compounds **1–3** were determined by spectroscopic analysis and by comparison of the spectral data with previously reported values. The cembranoid **3** was found to exhibit a moderate inhibitory effect on acetylcholinesterase.

## 1. Introduction

Alcyonarians are rich sources of sesquiterpenes and diterpenes with unique structural diversity and pronounced biological activities [1]. Among these, lobane and cembrane diterpenoids are typical of coelenterates of the orders Alcyonacea and Gorgonacea, which have been recognized as the most prolific sources of these classes of diterpenes. Lobanes, which have been isolated from the Gorgonian species *Eunicea fusca* [2] and from soft corals of the genera *Lobophytum* [3–6] and *Sinularia* [7–9], are known for their antibacterial, fungicidal and cytotoxic properties. Cembranes, found in several genera of soft corals, in particular *Lobophytum* [10], *Sinularia* [11] and *Sarcophyton* [12], are believed to play a role in the protection of soft corals from predators [13]. They have been reported to have antitumor, antimicrobial, anti-inflammatory, calcium antagonistic effects and neuroprotective activities.

In our search for bioactive substances from marine organisms, a biological screen of a collection of Comorian soft corals was initiated on toxic activity using brine shrimp *Artemia salina* assay [14] and on acetylcholinesterase inhibition using a TLC assay [15]. These two biological assays were chosen specifically because they are simple and rapid. The TLC bioassay gives quick access to the activity and its localization in complex matrices. The latter was also chosen because of the implication of acetylcholinesterase (AChE) inhibitors in the therapeutic approach to the fight against Alzheimer’s disease (AD).

AD is a progressive neurodegenerative disorder of the central nervous system that is characterized by an alteration of the cholinergic system and other neurotransmitter systems. One of the current therapeutic approaches to treating AD is aimed at restoring the native levels of acetylcholine in the central nervous system by using AChE inhibitors. Based on this strategy, AChE inhibitors are the most widely developed class of drugs approved for the symptomatic treatment of the disease. These include synthetic donepezil (Aticept®), rivastigmine (Exelon®) and the naturally occurring alkaloid galanthamine (Razadyne®) [16,17]. Some AChE inhibitors are known to have short half-time or strong side-effects. Even treatments with galanthamine, which is a potent long-acting drug with low toxicity, have shown a decline in clinical efficacy with time. The need for new potent selective and low side-effects AChE inhibitors is still enormous. After exploration of the terrestrial domain, marine organisms are being investigated.

**Figure 1:**
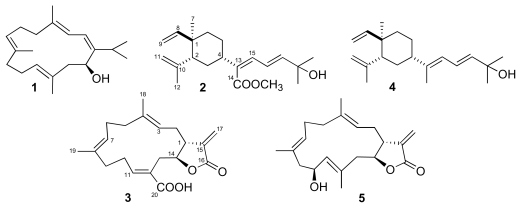
Structures of compounds **1**–**3**, fuscol (**4**) and crassumolide A (**5**).

Two specimens of soft corals were selected for their significant bioactivity against larvae of *Artemia salina* and acetylcholinesterase. Bioassays-guided separation resulted in the isolation of a known antitumor cembrane, (+)-sarcophytol-A (**1**), along with a new lobane, carbomethoxyfuscol (**2**) from a *Sarcophyton* sp. (family Alcyoniidae), and the isolation of a new cembranoid, crassumolide E (**3**), inhibitor of the acetylcholinesterase, from a *Lobophytum* sp. (family Alcyoniidae) (Figure 1).

## 2. Results and Discussion

Specimens of the soft corals *Lobophytum* sp. and *Sarcophyton* sp. were collected by scuba diving at the lagoon of Southern Mayotte, Comoros Island, northwest of Madagascar. All specimens were treated following the same procedure: the freeze-dried biological material was extracted with a mixture of methanol–dichloromethane 1:1 to give a crude extract. The latter was partitioned between ethyl acetate (EtOAc) and water, and the organic phase was subjected to a silica gel column eluted sequentially with hexane, dichloromethane, ethyl acetate and methanol. The EtOAc fractions of both soft corals were selected on the basis of their bioactivity, *i.e.*, the *Sarcophyton* EtOAc fraction showed toxicity against larvae of *Artemia salina,* and the *Lobophytum* EtOAc extract inhibited acetylcholinesterase. Fractions exhibiting activities were further chromatographied on silica gel column using different pentane–EtOAc mixtures. Final purifications were achieved by normal-phase HPLC to afford diterpenes **1**–**2** from *Sarcophyton* and **3** from *Lobophytum*.

### 2.1. Sarcophyton

Compound **1** was identified as (+)-sarcophytol-A by comparison of its spectral data with previously reported values [18]. The toxic activity against *Artemia salina* was mainly provided by this compound. Sarcophytol A has been reported to show antitumor activity [19] as well as potent inhibitory activities against several kinds of tumor promoters [20].

Compound **2** was isolated as a white solid, [α]^20^ _D_ +3.0° (c 0.86, CHCl_3_). Its molecular formula was established as C_21_H_32_O_3_ by HRESIMS analysis (*m/z* 355.2240 [M + Na]^+^, calculated for 355.2244). Its UV absorption (224 and 254 nm) indicated conjugation, and its IR spectrum revealed the presence of a hydroxyl group at 3400 cm^−1^ and an α,β-unsaturated carbonyl group at 1690 cm^−1^ besides further unsaturations and a terminal vinyl group (1630, 1450 and 895 cm^−1^).

**Figure 2:**
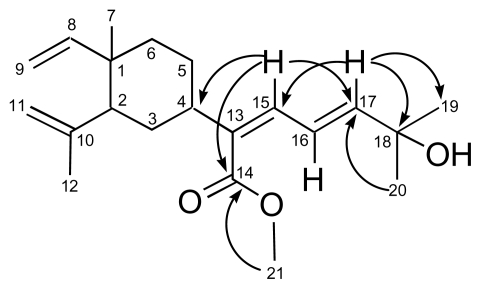
Key HMBC correlations in **2**.

The ^1^H and ^13^C NMR data were consistent with a lobane derivative (Table 1). In particular, the ^1^H spectrum revealed the presence of vinyl and isoprenyl systems of a lobane skeleton. In addition, three olefinic protons were observed in the downfield region at δ_H_ 6.05 (1H, d, *J* = 15.0 Hz, H-17), 6.33 (1H, d, *J* = 14.0 Hz, H-15), and 6.97 (1H, dd, *J* = 14.0 and 15.0 Hz, H-16), which suggested, according to COSY correlations between H-15/H-16 and H-16/H-17, the presence of a conjugated diene system. The large coupling constants (14.0 and 15.0 Hz) between H-15/H-16 and H-16/H-17 indicated the 13(*Z*),16(*E*) configuration of the diene system. The occurrence of two overlapping methyl signals at δ_H_ 1.36 (6H, s, H-19 and H-20) connected to one oxygenated carbon (δ_C_ 71.0, C-18) suggested that a hydroxyl group is located at C-18. Confirmation of this site was achieved by an HMBC experiment that unambiguously established the atom connectivities in **2** (Figure 2), *i.e.*, long-range correlations were found between the olefinic proton at δ_H_ 6.05 (H-17) and the quaternary carbon at δ_C_ 71.0 (C-18), and the methyl carbons at δ_C_ 29.6 (C-19 and C-20). Besides, long-range correlations were found between the methyl protons at δ_H_ 1.36 and C-17 and C-18. The sp^2^ quaternary carbon C-13 could be connected to a carbomethoxy group (δ_C_ 167.8, C-14; 51.6, C-21; δ_H_ 3.79, 3H, s, H-21) according to long-range correlation from H-15 to C-14. And a long-range correlation from H-15 to C-4 indicated that this quaternary carbon C-13 could be linked to the cyclic moiety. The presence of a carbomethoxy moiety was confirmed by the presence of the fragment ions at *m/z* 273 [M–COOCH_3_]^+^ and *m/z* 255 [M–COOCH_3_–H_2_O]^+^ in the mass spectrum. The conjugation of the diene with the ester function explained the significant differences in the chemical shifts of C-13, C-15, C-16 and C-17, as well as H-15, H-16 and H-17, compared to the ^1^H and ^13^C NMR data of fuscol (**4**), a lobane congener isolated from the gorgonian species *Eunicea fusca* [21] and from the soft coral *Lobophytum microlobulatum* [22], which present a methyl group instead of the carboxylate function.

The relative configuration of the various assymetric centers was deduced from the comparison with NMR data of diverse lobane congeners (the lobanes have all been described with the same stereochemistry as 1R, 2R, 4S); the similarity in the ^13^C chemical shifts of C-1 to C-12 in all compounds indicated the same relative configuration of the cyclohexane system. Thus, **2** was deduced to be methyl (1R*, 2R*, 4S*)-18-hydroxyloba-8, 10, 13(*Z*), 16(*E*)-tetraen-13-carboxylate.

Only one other publication mentions the coexistence of lobane and cembrane diterpenoids in a soft coral: fuscol and isofuscol were isolated with 11, 12-epoxycembra-1, 3, 7-triene, decaryol and 3, 4-epoxycembra-7, 11-dien-15-ol from *Lobophytum microlobulatum* [22]. The co-occurrence of lobane with sesquiterpenes (Δ^9(15)^-africanene [5, 23], 15-nor-13-keto-β-elemene [5, 21, 24]) or with steroids [5, 6, 21, 25, 26] has also been reported. Until now, lobanes have been exclusively isolated from the Gorgonian species *Eunicea fusca* [2] and from the soft corals of the genera *Lobophytum* [3–6] and *Sinularia* [7–9, 27]. It is the first time that a lobane derivative has been isolated from the *Sarcophyton* genus.

### 2.2. Lobophytum

Compound **3** was isolated as a white solid, [α]^20^_D_ +4.2° (c 2.12, MeOH). HRESIMS established the molecular formula as C_20_H_26_O_4_ (*m/z* 353.1727 [M+Na]^+^, calculated for 353.1724). The IR spectrum supported the presence of a γ-butyrolactone moiety (ν 1766 cm^−1^), and an α, β-unsatured carboxyl group (ν3500–2700 and 1683 cm^−1^), which was corroborated by an UV absorption maximum at 231 nm. ^1^H and ^13^C spectral data (Table 2) showed that **3** contained an α-methylene-γ-lactone [δ_H_ 2.97 (1H, ddd, *J* = 2.3, 4.3, 11.2 Hz, H-1), 4.11 (1H, ddd, *J* = 2.3, 5.1, 9.6 Hz, H-14), 5.67 (1H, d, *J* = 1.9 Hz, H-17a), 6.29 (1H, d, *J* = 1.9 Hz, H-17b) in the ^1^H NMR spectrum and δ_C_ 43.7 (d, C-1), 80.8 (d, C-14), 138.7 (s, C-15), 170.3 (s, C-16), 123.2 (t, C-17) in the ^13^C NMR spectrum], two methyl-bearing trisubstituted double bonds [δ_H_ 1.53 (3H, s, H-18), 4.84 (1H, dd, *J* = 4.0, 10.0 Hz, H-3), 1.58 (3H, s, H-19), 4.95 (1H, dd, *J* = 7.0, 8.0 Hz, H-7), and δ_C_ 15.5 (q, C-18), 122.0 (d, C-3), 137.6 (s, C-4), 17.0 (q, C-19), 124.0 (d, C-7), 133.9 (s, C-8)], and a conjugated olefinic system [δ_H_ 7.03 (1H, dd, *J* = 4.0, 9.0 Hz, H-11), δ_C_ 151.5 (d, C-11), 125.5 (s, C-12)]. The ^13^C NMR spectrum also disclosed six methylene carbons [δ_C_ 33.6, 39.1, 24.1, 37.5, 27.6, 31.7] and a carboxylic carbon at δ_C_ 172.4. The presence of a carboxylic acid was supported by the appearance of an additional signal at δ_H_ 12.3, characteristic of a carboxylic proton, in the ^1^H NMR spectrum of **3** recorded in DMSO-*d_6_*. Moreover, the presence of a conjugated carboxylic acid explained the low-field signal of H-11 at δ_H_ 7.03 assignable to the β-proton on the α, β-unsaturated carboxyl group.

**Figure 3:**
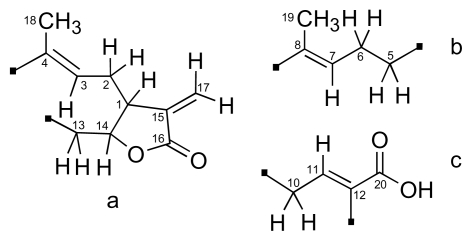
Partial structures for **3** based on ^1^H-^1^H COSY, HSQC and HMBC experiments.

**Figure 4:**
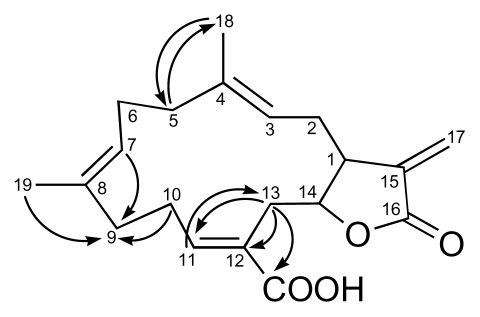
Key HMBC correlations connecting the three fragments in **3**.

After assignment of each direct C-H bonding based on HSQC data, the partial structures a, b, and c (Figure 3) were established by ^1^H-^1^H COSY analysis and supported by long-range HMBC correlations. The partial structures were reasonably connected to each other following HMBC correlations (Figure 4). The link between segments a and b *via* C-4 and C-5 was deduced from longrange correlations between H-5 to C-18. The connection between b and c *via* C-8 and C-10 was revealed by correlations between H-7, H-19, H-10a and H-10b to C-9. Finally, the connection between c and a *via* C-12 and C-13 was determined by correlations between H-13a and H-13b to C-11, C-12, C-20.

The *E*-configuration of the two methyl-substituted olefins was deduced from the ^13^C NMR data [δ_C_ 15.5 (q, C-18), 17.0 (q, C-19)] [28]. The *E*-configuration of the carboxylic-substituted olefin was suggested by the large downfield shifts of the vinylic proton (H-11) and carbon C-11 [δ_H_ 7.03 (H-11) and δ_C_ 151.5 (C-11)] [29]. In order to deduce the stereochemistry of the lactone ring junction, NOESY experiments have been recorded in different solvent conditions but no dipolar coupling could be observed between H-1 and H-14. According to literature [30–33], the coupling constant between the lactone methine protons (^3^*J*_1,14_) did not permit univocal stereochemical assignments due to the flexibility of the cembrane ring. However, previous studies tended to show that small coupling constants (ranging from 2.9 to 7.5 Hz) indicate a *trans*-fused γ-lactone ring while large coupling constants (from 6.5 to 9.3 Hz) suggest a *cis*-fused γ-lactone ring [33–36]. These propositions were done in agreement with several X-ray studies [29, 33, 37]. The ^3^*J*_1,14_ coupling constant of the lactone methine protons for **3** being 2.3 Hz, this suggests a *trans*-fused γ-lactone as in crassumolide A (**5**) [38], a cembrane isolated from *Lobophytum crassum*, whose structure is very similar. Thus, **3** was proposed as (1R*,14S*)-cembra-3(*E*),7(*E*),11(*Z*),15(17)-tetraen-14, 16-olide-12-carboxylic acid.

Although cembranes are the most commonly encountered class of secondary metabolites from *Lobophytum* soft corals, this new compound possesses an original functionality: an α, β-unsatured carboxyl group. This functionality is only present in four other marine cembranes: lobohedleolide and 7*Z*-lobohedleolide (isolated from *Lobophytum hedleyi* [27] and from *Lobophytum crassum* [34]), 2-epi-11*Z*-lobohedleolide (*Lobophytum* sp. [39]), and 17-dimethylaminolobohedleolide (*Lobophytum* sp. [10]). Its position in the cembane ring on C12 is unprecedented and the geometry of the C-11/C-12 double bond is unusual.

**Figure 5:**
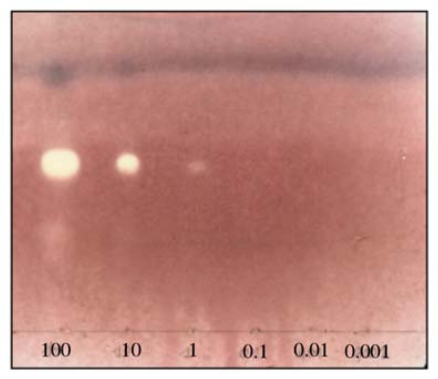
TLC plate showing the inhibition of acetylcholinesterase by **3** (100–0.001 μg applied). The assay was carried out using a Sigel 60 F 254 plate that had been eluted with hexane–ethyl acetate (70:30).

Moreover, this compound was found to exhibit inhibitory effects on AChE. A TLC bioautographic method for detection of acetylcholinesterase inhibitors was used for the screening of soft coral extracts. The active fraction of *Lobophytum* sp. was directly detected on the TLC. After purification on HPLC, the active fraction afforded compound **3**, that was further tested in order to establish the detection limit for its activity (Figure 5). **3** showed weak activity (1 μg at least) as AChE inhibitor, compared to galanthamine, an alkaloid isolated from plants of the Amaryllidaceae family (the smallest amount required 0.01 μg, cf. lit. [15]).

Only few AChE inhibitors have been isolated from marine organisms. One other terpene from the Alcyonacea family has been found to exhibit a modest AChE inhibition activity: cladidiol isolated from a *Cladiella* species [40]. Other examples are: a series of natural pigments with tetrazacyclopentazulene skeleton found in the zoanthid coral *Parazoanthus axinellae* (pseudozoanthoxanthins and zoanthoxanthins) [41], an irreversible inhibitor from the mollusk *Onchidella binneyi* (onchial) [42], a lipoidal metabolite isolated from the dinoflagellate *Gymnodinium breve* (BTX-B) [43], a nemertine alkaloid toxin (anabaseine) [44], a large 3-alkylpyridinium polymer isolated from the sponge *Reniera sarai* (poly-APS) [45, 46], a bromotyrosine-derived metabolite of an unidentified verongid sponge (aplysamine-4) [47] and spirocompounds isolated from the mediterranean sponge *Aplysina cavernicola* (aerothionin, homoaerothionin, 11, 19-dideoxyfistularin) [48], a diiodotyramine derivative from the Japanese gastropod *Turbo marmorata* (turbotoxin A) [49] and a pyridoacridine alkaloid from a Thai marine sponge *Petrosia* n. sp. (petrosamine) [50].

The biological interest of the newly isolated cembrane lies not only in its inhibitory effect on AChE, but also in the possibility of using this compound as a tool in the regulation of acetylcholine receptors. Over recent years, new results have emerged demonstrating that AChE inhibitors have additional pharmacological properties, *i.e.*, protection against neurotoxic agents, such as glutamate, and up-regulation of nicotinic acetylcholine receptors (nAChRs). Neuroprotection against toxic insults and use of nAChR modulators appears to be a promising new strategy against neurodegenerative diseases. Several tobacco and marine cembranoids have been found to interact with nAChRs in complex ways: as irreversible inhibitors at the agonist sites, as non competitive inhibitors, or as positive modulators. Studies of neuroprotection by a tobacco cembranoid and by the marine cembranoid sarcophytolide, isolated from the soft coral *Sarcophyton glaucum*, revealed apparent similarities between the two compounds, and allowed one to conclude that neuroprotection is based on a nicotinic mechanism [51]. It remains to be determined whether the AChR-mediated neuroprotection by those two cembranoids is the exception or a general property of most cembranoids. The isolation of new marine cembranoids with acetylcholinesterase inhibitory effects will give the opportunity to explore the specific action of cembranoids on the nicotinic acetylcholine receptors.

## 3. Experimental Section

### 3.1. General Experimental Procedures

TLC were performed on pre-coated TLC plates with Sigel 60 F 254 (Marchey–Nagel, Düren, Germany). HPLC was conducted with a MerckLiChrospher Si 60 (250 × 4 mm, 5 μm) column (Merck, Darmstadt, Germany), using different cyclohexane–EtOAc mixtures as eluent, and a refractometric detection. Optical rotations were measured at 20 °C on a Jasco P-1010 polarimeter. Ultraviolet (UV) spectra were recorded in methanol on a Perkin Elmer 551 spectrometer and Infrared (IR) spectra were obtained in chloroform on a Perkin Elmer 1600 FTIR spectrometer. ^1^H and ^13^C spectra, and 2D-NMR experiments were recorded on Brücker Avance DPX-250 NMR and Jeol EX 400 NMR spectrometers. Chemical shifts are given on a δ (ppm) scale with CHCl_3_ (^1^H, 7.26 ppm; ^13^C, 77.0 ppm) as internal standard. EI and HRESI mass spectra were acquired on an ATI UNICAM and a micromass LCT (ES) mass spectrometer, respectively.

### 3.2. Biological Material

Specimens of the soft corals *Lobophytum* sp. and *Sarcophyton* sp. were collected at the lagoon of Southern Mayotte, Comoros Islands, northwest of Madagascar, by scuba diving at a depth of 10 meters. Samples were identified by G. Williams (California Academy of Sciences). A positive identification could not be done, but the *Lobophytum* specimen showed similarities to *Lobophytum crassum* (Marenzeller, 1886), and the *Sarcophyton* specimen appeared close to *Sarcophyton infundibuliforme* (Tixier-Durivault, 1956). Voucher fragments are maintained at the California Academy of Sciences.

### 3.3. Extraction and Isolation

The freeze-dried samples of *Lobophytum* (780 g) and *Sarcophyton* (630 g) were extracted with CH_2_Cl_2_–MeOH 1:1 (500 mL × 4). The total extracts were evaporated under reduced pressure and partitioned between EtOAc and H_2_O. The organic fractions were taken to dryness (to provide 12.8 g for *Lobophytum* and 5.4 g for *Sarcophyton*) and subjected to chromatography on a methanol-desactivated silica gel column with hexane, dichloromethane, ethyl acetate and methanol. The EtOAc fractions of both soft corals were selected on the basis of their bioactivity, *i.e.*, the *Sarcophyton* EtOAc fraction showed toxicity against larvae of *Artemia salina* (at a concentration of 100 μg. mL^−1^), and the *Lobophytum* fraction inhibited acetylcholinesterase (at 100 μg. mL^−1^). Fractions exhibiting activities were further subjected to silica gel column (63–200 μm) eluted with different pentane–EtOAc mixtures. The *Sarcophyton* fraction, eluted with pentane–EtOAc 90:10 and active on *Artemia salina*, was purified by normal-phase HPLC using cyclohexane–EtOAc 95:5 as eluent, to afford 5.3 mg of **1**. The *Sarcophyton* fraction, eluted with pentane–EtOAc 70:30, was purified on silica-gel column (40–63 μm) with isochratic elution (pentane–EtOAc 80:20) then by HPLC (cyclohexane–EtOAc 95:5 as eluent), to obtain 5.0 mg of **2**. The *Lobophytum* fraction, eluted with pentane–EtOAc 60:40 and active on acetylcholinesterase, was purified by HPLC (cyclohexane–EtOAc 70:30 as eluent), to afford 240.0 mg of **3**.

*(+)-Sarcophytol-A* (**1**) was obtained as a colorless oil: [α]^20^_D_ +122.0° (c 0.38, CHCl_3_) (lit. +141.0°); ^1^H and ^13^C NMR data were in agreement with previously reported values [18].

Methyl (1R*,2R*,4S*)-18-hydroxyloba-8, 10, 13(E),16(E)-tetraen-13-carboxylate (**2**) was obtained as a white amorphous powder: [α]^20^_D_ +3.0° (c 0.85, CHCl_3_); UV (MeOH) λ_max_ (log ɛ) 224 (3.24), 254 (3.14); IR (CHCl_3_) ν_max_ 3400 br, 1690, 1630, 1450, 895 cm^−1; 1^H and ^13^C NMR, see Table 1; EIMS *m/z* (relative intensity) 289 (4.3), 273 [M–COOCH_3_]^+^ (16.0), 255 [M–COOCH_3_–H_2_O]^+^ (5.3), 197 (3.0), 173 (5.3), 161 (13.8), 147 (26.4), 135 (45.0), 121 (100.0), 109 (85.6), 95 (78.8), 91 (71.3); HRESIMS *m/z* 355.2240 [M+Na]^+^ (calculated for C_21_H_32_O_3_Na, 355.2244).

(1R*,14S*)-cembra-3(E),7(E),11(E),15(17)-tetraen-14, 16-olide-12-carboxylic acid (**3**) was obtained as a white amorphous powder: [α]^20^_D_ +4.2° (c 2.12, MeOH); UV (MeOH) λ_max_ (log ɛ) 230 (3.12); IR (CHCl_3_) ν_max_ 3500–2700, 1766, 1683 cm^−1; 1^H and ^13^C NMR, see Table 2; EIMS *m/z* (relative intensity) 312 [M–OH]^+^ (1.3), 285 [M–COO]^+^ (7.9), 256 (3.6), 239 (11.0), 149 (18.7), 141 (27.0), 135 (65.0), 121 (59.0), 107 (100.0), 95 (47.6), 91 (52.0); HRESIMS *m/z* 353.1727 [M+Na]^+^ (calculated for C_20_H_26_O_4_Na, 353.1724).

### 3.4. Brine Shrimp Lethality Test

Eggs of *Artemia salina* (Dohse, Aquaristik GmbH, Bonn, Germany) were hatched in a small tank filled with natural sea water at 25 °C under continuous light regime. After 48 h, the phototropic nauplii were collected; 10 shrimps were transferred to each sample vial using a pipette, and seawater was added to a final volume of 180 μL. 20 μL of each sample (solution of 1 mg. mL^−1^ in water and 10% EtOH) were added in the 96-well plate. The toxicity was determined after 24 and 48 h of exposure. The experiments were done twice in duplicate.

### 3.5. Acetylcholinesterase Inhibition Test

Acetylcholinesterase (Sigma, St Louis, USA; 1000 U) was dissolved in 150 mL of 0.05 M Tris-hydrochloric acid buffer at pH 7.8, with 150 mg of bovine serum albumin (Merck, Darmstadt, Germany). After deposition of the samples on TLC (100 μg) and migration in a suitable solvent, the TLC plate was dried to remove the solvent, sprayed with the enzyme solution and dried again. The plate was placed in an incubator at 90% humidity and 37 °C for 20 min. For detection of the enzyme, 10 mL of a 1-naphtylacetate solution (250 mg in 100 mL of ethanol) and 40 mL of a Fast Blue Salt solution (400 mg in 160 mL of distilled water) were mixed and sprayed onto the plate to give a purple coloration after 1–2 min. Inhibitors of acetylcholinesterase appear as white spots.

## Figures and Tables

**Table 1:** ^1^H and ^13^C-NMR data^a^ of compound **2** in CDCl_3_

Atom n°	^1^H NMR	^13^C NMR^c^		COSY	HMBC
1		39.8	Cq		
2	2,20 m	52.7	CH	3	
3	1,56 m	33.4	CH_2_	2, 4	
4	2,02 m	41.5	CH	3	
5	1,60–1,57 m^b^	27.2	CH_2_		
6	1,50–1,45 m^b^	39.7	CH_2_		
7	1.08 s	16.6	CH_3_		8
8	5.82 dd (11.0, 18.0)	150.0	CH	9a, 9b	7
9a	4.91 d (18.0)	110.0	CH_2_	8	1, 8
9b	4.90 d (11.0)			8	1, 8
10		147.4	Cq		
11a	4.82 br s	112.3	CH_2_		2, 12
11b	4.58 br s				2, 12
12	1.70 s	24.8	CH_3_	11a, 11b	10, 11
13		136.8	Cq		
14		168.7	Cq		
15	6.33 d (14.0)	134.3	CH	16	4, 14, 17
16	6.97 dd (14.0, 15.0)	123.6	CH	15, 17	
17	6.05 d (15.0)	146.7	CH	16	15, 18, 19/20
18		71.0	Cq		
19	1.36 s	29.6	CH_3_		17, 18
20	1.36 s	29.6	CH_3_		17, 18
21	3.79 s	51.6	CH_3_		14

a ^1^H NMR, 400 MHz; ^13^C NMR, 100 MHz; chemical shifts are given in δ values relative to CHCl_3_ as internal standard and coupling constants in Hz are in parentheses.

b These protons appears as very broad multiplets in the proton spectrum. H_2_-5 and H_2_-6 data may be interchanged.

c Carbon atom multiplicity was deduced by DEPT.

**Table 2:** ^1^H and ^13^C-NMR data^a^ of compound **3** in CDCl_3_

Atom n°	^1^H NMR	^13^C NMR^b^		COSY	HMBC
1	2.97 ddd (2.3, 4.3, 11.2)	43.7	CH	2a, 2b, 17a, 17b	
2a	2.18 m	33.6	CH_2_	1, 2b, 3	1
2b	2.25 m			1, 2a, 3	
3	4.84 dd (4.0, 10.0)	122.0	CH	2a, 2b, 18	18
4		137.6	Cq		
5	1.95 m	39.1	CH_2_	6	6, 18
6	2.15 m	24.1	CH_2_	5, 7	5
7	4.95 dd (7.0, 8.0)	124.0	CH	6, 19	9, 19
8		133.9	Cq		
9	2.22 m	37.5	CH_2_		
10a	2.30 m	27.3	CH_2_	11	9
10b	2.50 m			11	9
11	7.03 dd (4.8, 9.0)	151.5	CH	10a, 10b	13, 20
12		125.5	Cq		
13a	2.57 m	31.7	CH_2_	13b, 14	1, 11, 12, 14, 20
13b	2.68 m			13a, 14	1, 11, 12, 14, 20
14	4.11 ddd (2.3, 5.1, 9.6)	80.8	CH		2, 16
15		138.7	Cq		
16		170.3	Cq		
17a	5.67 d (1.9)	123.3	CH_2_		1, 16
17b	6.29 d (1.9)				1, 15, 16
18	1.53 s	15.5	CH_3_	3	3, 4, 5
19	1.58 s	17.0	CH_3_	7	7, 8, 9
20		172.4	Cq		

a ^1^H NMR, 400 MHz; ^13^C NMR, 100 MHz; chemical shifts are given in δ values relative to CHCl_3_ as internal standard and coupling constants in Hz are in parentheses.

b Carbon atom multiplicity was deduced by DEPT.
